# Strategic management of the health workforce in developing countries: what have we learned?

**DOI:** 10.1186/1478-4491-5-4

**Published:** 2007-02-26

**Authors:** Scott A Fritzen

**Affiliations:** 1 LKY School of Public Policy, National University of Singapore, 469C Bukit Timah Road, 259772, Singapore

## Abstract

The study of the health workforce has gained in prominence in recent years, as the dynamic interconnections between human resource issues and health system effectiveness have come into sharper focus. This paper reviews lessons relating to strategic management challenges emerging from the growing literature in this area. Workforce issues are strategic: they affect overall system performance as well as the feasibility and sustainability of health reforms. Viewing workforce issues strategically forces health authorities to confront the yawning gaps between policy and implementation in many developing countries.

Lessons emerge in four areas. One concerns imbalances in workforce structure, whether from a functional specialization, geographical or facility lens. These imbalances pose a strategic challenge in that authorities must attempt to steer workforce distribution over time using a limited range of policy tools. A second group of lessons concerns the difficulties of central-level steering of the health workforce, often critically weak due to the lack of proper information systems and the complexities of public sector decentralization and service commercialization trends affecting the grassroots.

A third cluster examines worker capacity and motivation, often shaped in developing countries as much by the informal norms and incentives as by formal attempts to support workers or to hold them accountable. Finally, a range of reforms centering on service contracting and improvements to human resource management are emerging. Since these have as a necessary (but not sufficient) condition some flexibility in personnel practices, recent trends towards the sharing of such functions with local authorities are promising.

The paper identifies a number of current lines of productive research, focusing on the relationship between health policy reforms and the local institutional environments in which the workforce, both public and private, is deployed.

## Review

Having long suffered "woeful neglect," [1] the study of the health workforce is enjoying increased interest in recent years, to judge by the growing range of research being conducted across a variety of sub-fields. This paper plumbs recent work on the health workforce for insights into one of its important, though often implicit, cross-cutting themes: the need for, and current weaknesses in, the *strategic management *of the health workforce in developing countries. After exploring the nature of 'strategic' management in the health sector, I group ten lessons into four areas: workforce structure, the 'steering' of the workforce, drivers of behavior at the facility level and approaches to improving the performance of human resources. The paper also sketches questions likely to preoccupy researchers in this area in the coming years.

### What is 'strategic' about the health workforce?

One conclusion of a growing number of case studies and policy analyses of the health workforce concerns the *strategic *nature of health workforce issues, in several senses. The workforce, arguably the most important input to any health system, has a strong impact on overall health system performance [2,3]. Health sectors in developing countries have faced a wide variety of systemic pressures in recent years. Although the specifics vary by country setting, these have often included pressures towards marketization of health services [4], civil service restructuring [5], decentralization [6,7] and an overall trend towards increasing geographical and socioeconomic disparities in many countries. The feasibility and sustainability of reforms introduced in these systems relies heavily on the level of 'buy-in' and well-being of health sector personnel at all levels. As West put it in her review of the National Health System in Britain: "The quality of patient care may be related in an important way to the quality of life experienced by staff at work" [8].

The health workforce is also strategic in that almost any type of desired reform outcome depends on specific changes in *workforce behavior *throughout the system, and such behavior is profoundly affected by a range of factors that are not directly under the control of central and local authorities. Understanding and changing negative phenomena at the local level – such as illegal user charges or drug overprescription – depends on an understanding of the effective incentives faced by grassroots service providers. Studying how health personnel are likely to respond to new rules, roles, responsibilities and resources is thus essential for gauging the feasibility of reform interventions.

'Strategic' management implies a more coordinated, systematic policy approach to the workforce based on a clear situation analysis and linked to at least a medium-term vision of desired outcomes for the health sector as a whole [9,10]. Constraints on strategic management, which is both capacity- and strategy-intensive, often stem in developing country settings from particularly short policy horizons and weak implementation capacities [11,12].

While the literature on the health workforce is still poorly developed in relation to other aspects of health care, a range of case studies in recent years has yielded valuable insights regarding patterns and challenges of strategic management in the sector. Ten lessons stand out.

### Structural aspects of the health workforce

#### 1. Imbalances in workforce structure are both a symptom of underlying problems and a cause of poor health system performance

Several recent studies have cast new light on the 'old' problem of imbalances in the health workforce [13-16]. One contribution has been to systematic categories of imbalances, including:

• *Overall supply/demand: *In theory, workforce numbers should be determined by specific objectives set by policymakers for the health sector and by the demand for health services. In practice, a number of factors – such as variations in the historical production of the workforce, and the long time lag between training and deployment of health personnel – can intervene. It is commonly thought that the public health sectors of many countries suffer from a surplus of workers who are not particularly productive; hence a focus in a number of countries on downsizing the labor force.

• *Profession/specialty: *Imbalances in this area concern the distribution of doctors, nurses, paraprofessionals and other health personnel. One generalization from the public health literature is that systems often underinvest in the production of paraprofessionals, nurses and assistant doctors relative to the expensive education of doctors [16].

• *Geographic: *Developing countries are often said to suffer from 'urban bias' – a situation in which the political and economic forces of a country reinforce provision of services and investments in urban areas, reinforcing disparities in access to health services and in health outcomes [17]. This can also influence the distribution of health personnel away from rural and remote areas that tend to be poorer. However, there may be a tension between concentrating staff in more densely populated areas (which may be better off) as opposed to poorer remote areas. This reflects a broader trade-off often encountered between equity and efficiency in health care.

• *Institutional imbalances: *These include the question of how to deploy staff between curative and preventive functions of the health sector, as well as their distribution at various tiers and facilities of the system [13].

• *Public/private imbalance: *Most systems have some mix of public and private personnel, and countries are arrayed somewhere along a continuum in terms of their distribution among these poles. To imply that there is an imbalance is to say that some important function of the health system is being underperformed because of the distribution of personnel along this pole. A typical example would be public health missions that are suffering from a lack of trained personnel or resources [14].

• *Gender and ethnic imbalances: *Since the Alma Ata summit which gave a major boost to the concept of primary health care, a key idea has been that the health sector should be democratic, and the health personnel should be representative of the population. An imbalance in this area would suggest that workforce demographics fail to reflect important variations (in ethnicity or gender) of the client populations, with the implication that such populations are being underserved [13].

The notion of "workforce imbalance" is a subjective appraisal; there is no inherent equilibrium point for any of the variables above. That will depend on context, objectives and values. The broader, strategic issue concerns the means health authorities have at their disposal for intervening in order to affect workforce imbalances. Such means are often considerably weaker than authorities realize, not least due to an overreliance on administrative norms and centrally dictated quotas for workforce production and deployment, often badly out of alignment with transitioning, decentralizing countries [18].

### Shifting roles of the center and local governments in health workforce issues

The issue of the health workforce is complex partly because of the great number of actors and stakeholders involved. Much of the literature on the workforce deals with the issue of the mix of roles and responsibilities between different levels of government, which have been in some transition due to decentralization.

#### 2. Capacities for strategic workforce planning in Ministries of Health are often critically weak

Information systems relating to the workforce are typically sketchy [19], and planning is technical rather than strategic in nature, [20] for instance leaving "key questions about the distribution, qualifications, motivation, development, and performance of staff unexplored." [21] One consequence of this is that central-level steering of the health workforce and the behavior of personnel at local levels is often surprisingly weak, as noted above [5,22]. If the primary instruments employed by the central level to steer the health system are laws, regulations and administrative norms, the literature notes large gaps between policy and local implementation. A study of China's health sector highlights the "difficulties involved in efforts to influence provider behavior through a national level legislative framework in a situation of decentralization of control over those providers, due to extreme regional variation in economic situations and limited resource inputs from the centre" [23]. Such difficulties are highlighted when, as in China, only some 30% of health worker salaries is paid by the central government [23].

The balancing act Ministries of Health must walk is between being overly prescriptive (and thus developing rules that are inappropriate for particular local conditions) and issuing guidelines too general to be of use. Where the policy-implementation gap is overcome, it is usually due to the development of "specific guidelines developed by organizations of health professionals or other advisory bodies" for the implementation of health laws and policies "to bridge the gap between legal theory and everyday practice" [24].

#### 3. Provinces can potentially play an important role in human resource management, but they are often constrained by overly centralized health systems

The ability of provinces and local governments in general to contract, hire, promote and fire human resources is a major determinant of the flexibility of any health system and on the viability efforts to promote improved responsiveness (the "short route to accountability", in the World Bank's phrase [25]) between local service providers and the end users of services.

Yet, as suggested by Table 1, taking several countries in East and Southeast Asia as a sample, control over hiring and firing of workers is rather limited in a number of countries, even those with fairly advanced stages of decentralization (such as Indonesia and the Philippines). Some countries in which donors play a strong role (as in Cambodia) have introduced greater flexibility in local government contracting of health staff, something that "provides the opportunity for signatories to negotiate mutually agreed activities, funding and outputs for specific health programmes" [20]. Yet decentralization of such powers can lead to health workforce "fragmentation", and can pose a "threat to workers' well-being" if it negatively affects (as in Uganda) professionalism, career mobility and the timeliness of salary payments" [27]. Thus some analysts point towards a "tension between the objective of increasing efficiency and local government autonomy, on the one hand, and the quality and equity benefits of a uniform national service cadre with vertical mobility, on the other" [6].

**Table 1:** Sub-national influence in human resource management in East Asia

Domain	Cambodia	China	Indonesia	Philippines	Thailand	Vietnam
Budget control						
• Determine the wage envelope	*	*	*	**	*	*
• Dismiss surplus staff		**	**	***		*
Establishment control						
• Control overall staffing numbers	*	**	*	**	*	*
• Control staffing numbers in individual facilities		**	**	***		**
Recruitment						
• Formal employer	***	***	*	***	**	*
• Have authority to hire	**	***	**	***	*	***
• Have independent recruitment mechanism	*	**	**	*	*	*
Career management						
• Promotion is available	*	**	**	***	*	*
• Transfers within local government are possible	*	*	***	***	*	**
Performance management						
• Direct and supervise activities	***	***	**	***	***	***
• Conduct evaluations	*	***	**	***	*	*
• Offer financial rewards	*	*	**	**	*	**
• Discipline and fire underperforming staff	*	*	**	**	*	**
Pay policy						
• Set overall wage rates	*	*	*	*	*	*
• Set local incentives/salary top-ups	**	***	**	***	*	**

*** = full; ** = partial; * = no subnational control, focusing on the strongest subnational level of government for each country. Data underlying the assessment range from 2000 – 2005.

Source: adapted from table 7.3 in [26] supplemented by author's estimates for Vietnam.

### Worker capacity, motivation, and performance

The academic literature on the workforce focuses much attention onto the linkages between the conditions in which health personnel work and their performance. One useful way of thinking about how to boost workforce capacity to perform at a high level is to think of what the workforce 'can do' (what skills and training enables people to do) and what the workforce 'will do' (feels motivated and empowered to do). Such an institutional analysis is essential to understanding policy implementation gaps in the sector.

#### 4. On the 'can do' side, workers are often unprepared to take on their new functions in more complex systems, especially when these systems are decentralizing

Typical training of health personnel emphasizes factual, specialist medical knowledge. But much of what public personnel actually must do to fulfil their functions involves higher-order analysis, supervision and inspection, coordination across multiple actors (including both local authorities and communities) and a range of managerial tasks [21]. Table 2 gives one example of tasks assigned typically to district-level health managers. The literature notes that district managers, in particular, feel and in reality are poorly supported in many of these functions. District health managers are often found to be particularly weak in systems management (community involvement and intersectoral co-operation), monitoring activities and the systematic organization of meetings. One study notes that district managers are "rarely involved in the identification of priority health problems or of high-risk groups, and fail to use health service indicators sufficiently for the analysis of the district health system" [28].

**Table 2:** Common tasks of district-level health managers

***Task category***	***Examples***
Needs assessment and planning	Analysis of context, health, health service and priority setting
Resource management	Human resources (selection, team management and communication, management of conflict, financial resources, facilities management (drugs, acquisition and maintenance of equipment), supervision of grassroots health workers
Systems management	Dissemination and interpretation of new health policies; development of practical implementation guidelines or measures for local area Intrasectoral collaboration (including referral systems) Intersectoral collaboration (e.g. between health facilities and schools) Social mobilization and community participation Regulation/coordination with private sector providers to address unexpected problems or regular campaigns
Monitoring and evaluation	Quality assurance, inspections, regular reporting to upper levels

Source: Adapted from [28]

Part of the complexity of the managerial environments in which health personnel must function comes from the multiple sources of accountability to which they are subject [27]. Managers and personnel at the grassroots must balance the demands of "those responsible for specific policies, such as reproductive health policies, and those responsible for managing the integrated delivery of all policies, with their resultant contestations over authority and resources" [29]. They must also manage the increasing commercialization of services that is an important feature of many developing country health sectors [4,30].

#### 5. On the "will do" side of worker motivation, the literature emphasizes the importance of considering a broad range of factors that affect worker motivation

**Figure 1:**
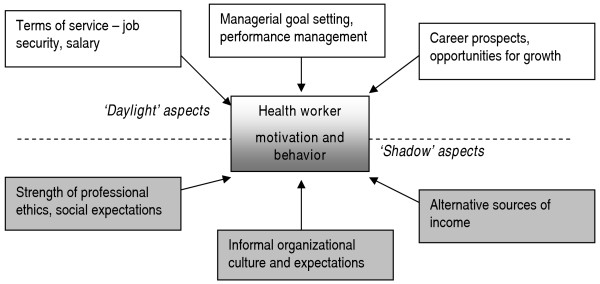
Factors affecting worker motivation and behavior.

These can be understood in terms of metaphorical 'daylight' and 'shadow-side' factors, distinguished by the degree to which they are amenable to intervention by the health authorities (and easily accessible to researchers). Both obvious "daylight" factors such as worker terms of service and managerial supervision can affect worker motivation and behaviour; but the hidden or 'shadow' side of organizational life and worker motivation must also be recognized (see figure 1). It includes the value sets of workers (which are influenced both by professional norms, social ideas about the professional roles workers are playing and by the broader organizational culture of the civil service).

The shadow side also includes the availability of livelihood strategies and alternatives. Whether health workers are 'over-' or 'under-paid,' relative to some external standard – and debate on how to approach this issue continues in the literature [31] – is not the sole point. Rather, health workers, like all other workers will gauge the need and possibility of earning additional income (including any risks associated with it) when considering the effort they put into discharging their official responsibilities. Of course, the shadow and daylight sides are interlinked:

...confirmation and even salary are forms of recognition as well as ways of satisfying lower order needs such as survival and security. But environmental factors may affect which order (lower level survival needs or higher level desire for recognition) is most prominent. The poorer the satisfaction of basic terms of service factors, the less likely higher order incentives are to be central [32].

#### 6. On both "can" and "will do" measures, the institutional environments in which many health workers work can be more disabling than enabling

Incentives for positive performance of the health workforce are reported to be very weak across a range of developing country health sectors, both from the 'daylight' and 'shadow' sides of the health facility environment.

On the 'daylight' side, workers may feel they have little to gain from working hard or being responsive to either their clients or superiors. Poor career paths and promotion opportunities lead to health workers feeling 'stuck', while official salaries often cover only part of a worker's needs or overall income (given alternative livelihood strategies, such as engaging in part-time private sector health services or entirely different informal occupations) [33].

Also on the 'daylight' side, a range of functions related to both performance management (by which is meant the systematic communication of job expectations followed by regular performance reviews) and strategic personnel planning ("the creation of new types of jobs, re-profiling of old ones or the addition or abolishing of staff positions in accordance with need") [34] – is found to be weak or typically non-existent [35]. The Cambodian case is typical: "sub-national units have no effective system of performance management for individuals or work units with no system for linking individual performance to the goals and functions of the organization or for rewarding appropriate initiatives and behaviour"[20]. Part of the reason performance management is rare in public services in developing countries is that "its prerequisites (such as a living wage for health workers, and the availability to them of drugs, equipment and transport) are often missing" [34]. When the health sector is severely underresourced it is difficult to hold people accountable for how they do their jobs. Also, workers often feel disempowered by the narrow range of authority they are granted in conducting their jobs and by their lack of consultation regarding major reform efforts affecting their jobs. Even in systems undergoing significant administrative decentralization, there is often a disjunction between formal responsibilities and requisite resources to meet minimum specified standards – a classic recipe for workforce frustration and for the failure of decentralization reforms [36].

The 'shadow' side of organizations is also often found to undermine morale in the health sector. The informal norms and work ethics of frontline civil servants in more remote localities can be oriented more towards alternative livelihood strategies and "muddling through" than they are towards high standards of service delivery [27]. A sense of professionalism can be difficult to maintain especially in remote areas, particularly where staff feel the commitment of central and local authorities to the sector, and social expectations regarding their performance, are weak [37].

#### 7. The collective impact of weak institutional environments at the facility level can be a strong determinant of health sector performance

Studies note that even in well-resourced facilities, doctor training and experience tend to be weakly correlated with the quality of care provided, which is taken by analysts to mean,

... not that physicians are unimportant for quality but that organisational context is far more important in setting limits (upper and lower) for physicians than formerly recognised. Medical staff organisation– including peer review, selection and continued review of new staff members, and participation in policy making committees–have also been shown to be positively related to quality of patient care [8].

### Approaches to boosting workforce performance

#### 8. Despite the difficulties noted above, the literature identifies a number of characteristics of high-performing health facilities and personnel

Such organizations share the following characteristics [38].

• A strong sense of mission and sense of commitment to that mission by staff.

• A relatively high level of prestige and social status accorded to those who work in the organization.

• A culture oriented towards results both individually and organizationally. All members of the group are evaluated against performance objectives regularly and are expected, both by managers and by co-workers, to pull their weight; and the organization itself constantly evaluates its performance against external objectives and benchmarks.

• Lines of feedback from the end users of services are open and actively used to improve service delivery.

What is striking in the literature is the notion that *organizational culture *can be as critical as the direct monetary incentives of the workforce [39]. While pay and job security are clearly important determinants of morale, many organizations in developing countries have been able to significantly improve performance by cultivating a participatory, open, performance-oriented culture in which the workforce is deployed.

The literature also stresses that allowing "some autonomy in personnel matters" is an essential facilitative condition for developing the organizational performance culture noted above [38]. A review of high-performing public organizations in developing countries found that they enjoyed autonomy to "identify positions, advertise for candidates, establish routines for hiring people to fill positions, promote people on the basis of organizationally defined standards and priorities, and punish those who did not meet these standards" [38]. Recent trends towards the sharing of some personnel management functions with local authorities and facility managers (as shown in Table 1) are promising in this regard, although a number of enabling factors will be necessary to support and reorient such functions [26].

There is no doubt that it is possible to improve health sector performance in developing countries using these reform approaches, as attested by several documented case studies [11]. However, the health sector has some special challenges that make it important to apply reform efforts with care, as attested in the final two points addressing popular approaches to health sector reform.

#### 9. Decentralization is no panacea for the health sector; it will not strengthen accountability and performance unless factors supporting high performance are put into place

Decentralization has been in vogue in developing countries for over two decades, but there is no evidence in the growing literature on health sector decentralization that decentralization reliably increases sector performance [6,40]. A review of TB control in Nepal concludes that decentralization

...can lead to inequity, political manipulation, fragmentation, increased bureaucratic costs and the overall weakening of the public sector...and affects the incentives and career prospects of health staff. This complexity raises serious questions regarding the 'why', 'what' and 'how' of decentralisation [41].

The important lesson in managing the process of health sector decentralization is "to ensure that the newly empowered organization is required to deliver clearly identifiable and measurable objectives. At the same time the organization is given the necessary resources and discretion over their use, to permit these objectives to be met" [42]. The conceptual shift that is required is to examine what approaches and parts of decentralization work in which local contexts, and to adjust the decentralizing reform to match this [36].

#### 10. Approaches to improving performance that focus on increasing competitive pressures on health facilities run the risk of undermining core public health functions and values

The curative and preventive sides of public service provision typically cannot be delinked; the incentives of providers who often must attend to both sets of functions are affected by increasing pressures in the area of curative care [17]. It is in general hypothesized in the literature that 'what gets measured gets done', and even more so 'what gets paid gets done'; thus, if the material incentives shift towards curative health provision, it is likely that managerial and staff attention will be focused there as well. An overemphasis on revenue generation often leads to declining access by the poor as well as overproduction of health services. This effect may be particularly pronounced in transition settings such as China and Vietnam [18,30,43,44]; one case study in China found that the introduction of output-related bonuses for doctors increased "unnecessary care," drug sales and inpatient admission rates [45].

Moreover, in most countries the curative and preventive sides of health financing are linked in multiple ways. For example, in a decentralized, commercialized health sector, implementation support for preventive functions may be increasingly financed out of local government budgets (which can vary dramatically) or even facility-level revenues [43]. This may accentuate disparities between facilities that are capable of such supplementation and those that are not.

Competitive pressures may be part of the reform mix. The move to contract out service provision, even for preventive health functions such as health information, to providers who are paid on the basis of concrete outputs and outcomes is one example of a promising approach [46]. But taken as a whole the literature can hardly be said to be optimistic.

## Conclusion

**Figure 2:**
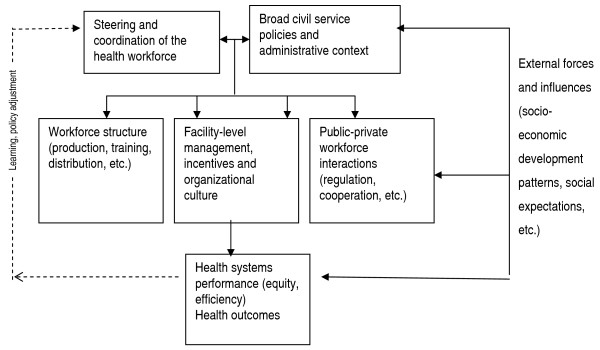
Dimensions of health workforce research.

Figure 2 lays out some of the central components of research concerning the strategic management of health workers in developing countries. It highlights core dimensions of workforce issues, corresponding roughly to three types of policy instruments that health policymakers have at their disposal to manage the workforce 'strategically':

• Structural issues such as the recruitment and distribution of workers (policy instruments: direct investment in the production of the workforce and regulations governing their distribution);

• Management and motivation issues (policy instruments: organizational reforms, management and supervisory patterns and workforce terms of service); and

• Private sector environment (policy instruments: regulation and standard setting).

In addition to emphasizing this menu of policy choices, the framework is meant to highlight the external factors that impact on actual performance. 'External', from a health policymakers' perspective, includes both policies that originate from outside the sector – such as decentralization and public administration reform – and changing social expectations and development levels and patterns, including increased disparities throughout a country. The combined effect of these factors, including on that upon the workforce, leads to overall health sector performance, which eventually leads to particular health impacts (although these are not directly addressed here). Ideally, the system should be able to learn over time how to adjust policies and projects to achieve a more positive impact. Important questions on which some productive areas of current research on strategic workforce management are likely to continue can be situated in the figure. For example:

• What kinds of impacts are environmental and policy changes originating outside the sector having on the health workforce?

• What do these changes imply for effectiveness of different mechanisms and interventions employed by health authorities to steer and coordinate workforce issues?

• How can the health sector take advantage and avoid the risks of public sector decentralization trends? How will the roles of the central-level authorities in workforce management be changing in this process?

• How can we assess facility-level determinants of motivation and high performance, including the impact of various types of accountability mechanisms, in order to influence workforce motivation in ways that can be implemented in highly resource-constrained environments?

• What effect are private sector forces having on the set of incentives of the public health workforce? Furthermore, how can the private sector workforce itself be more effectively regulated and made to synergize to the maximum extent possible with that of the public sector?

Three additional aspects of the figure deserve mention. First, policymakers control some but not all of the levers necessary to improve health sector performance. In addition to policies, the broader institutional environment affecting local service delivery is important to consider. Second, policies can have both intended and unintended consequences on the workforce. For instance, efforts to increase competitive pressures by boosting managerial and financial autonomy of facilities can have unintended side-effects on workforce performance; these are important to analyze. Finally, the impacts of any health sector reform in large, heterogeneous countries are likely to be mixed, and a key focus of health analysts should be on the attempt to explain the resulting patterns.

Efforts to study the health workforce will likely continue to gain in prominence, and are likely to figure more prominently in the next ten years than they have in recent decades. Within the growing number of case studies and analytical reviews, the issue of what makes for effective *strategic *management of the health workforce deserves continued attention, particularly in developing country settings.

## Competing interests

The author(s) declare that they have no competing interests.
